# Hidden defensive morphology in rotifers: benefits, costs, and fitness consequences

**DOI:** 10.1038/s41598-017-04809-z

**Published:** 2017-07-03

**Authors:** Xuwang Yin, Wen Jin, Yanchun Zhou, Peipei Wang, Wen Zhao

**Affiliations:** Liaoning Provincial Key Laboratory for Hydrobiology, College of Fisheries and Life Science, Dalian Ocean University, Dalian, China

## Abstract

To cope with predation, many prey species have developed inducible defenses in terms of morphology, behavior, and life history. Rotifers were the first model organisms used to evaluate the ecology and evolution of inducible defenses in aquatic ecosystems. Since the middle of last century, only visible morphological defenses, such as spine development, have been found and only in a few rotifer species. Given the development of ultrastructural defenses is taxonomically widespread in aquatic ecosystems, we hypothesize that rotifer prey, particularly small-sized species, can develop such inducible defenses. We evaluated morphological response of two common *Brachionus* herbivores (*B*. *calyciflorus* and *B*. *angularis*) to predatory rotifer *Asplanchna brightwellii*. Results confirmed existence of predator-induced ultrastructural defenses, which are expressed as increased lorica thickness and enhanced lorica hardness. Such inducible defenses are more evident and effective in the smaller sized *B*. *angularis*, leading to higher fitness of *B*. *angularis* in predator-prey interactions. As anticipated, development of defenses has inevitable fitness costs manifested as decreased reproduction or reduced sex investment. Our results not only extend understanding of inducible ultrastructural defense to other planktonic taxa that were previously observed only in cladocerans, but also verify effective mechanical protection of such hidden defensive morphology.

## Introduction

Predation is an important selective agent driving the evolution of prey species. To alleviate the tremendous effects of predation on survival community dynamics, many prey species have developed inducible defenses to impede or prevent capture and ingestion by a variety of predators^1, 2^. Inducible defenses, as striking examples of phenotypic plasticity^3^, can evolve because the presence of some reliable cues released by predators induces several kinds of effective but costly defenses that help prey species survive in a variable and unpredictable predation risk environment^4^. Many freshwater organisms, e.g., algae, rotifers, cladocerans, and amphibians, have developed various inducible defenses, including defensive morphology, diel vertical migration, swarming, life history shift, and diapause, to avoid predation^5–7^. Inducible defenses can not only generate a type of adaptive prey mechanism to defend against predation, but also promote coexistence of prey species and constitute community stability^8, 9^.

Monogonont rotifers, as an important part of zooplankton communities, were the first model organisms used to investigate the ecology and evolution of inducible defenses in aquatic ecosystems^4, 10^. Nearly 1,600 monogonont species exist worldwide^11^; however, since the discovery of inducible defenses in rotifers^12, 13^, only fourteen rotifer species in the genera *Brachionus*, *Keratella*, *Plationus*, and *Filinia*, have been reported to exhibit predator-induced defenses^10^. Almost all identified inducible defenses of these rotifers are morphological, involving the development and elongation of lorica spines^10^. Inducible defenses are common in freshwater ecosystems^1, 5–7^, hence the lack of evidence to determine the widespread existence of inducible defenses in rotifers has been confusing scientists^14^. At present, no satisfactory explanation for this phenomenon has been proposed.

In aquatic ecosystems, some species can change structures of cuticle under predation risk, e.g., increased thickness and rigidity of *Daphnia* carapace in response to the predator*y* tadpole shrimp^15^, increased cuticle thickness of the dragonfly *Leucorrhinia* larvae in response to fish^16^, and increased shell thickness of the snail *Physa* in response to crayfish^17^. Ultrastructural defenses are predicted to be adaptive because it might be difficult for predator to crush and ingest the prey with rigid cuticle^18, 19^. In general, rotifers are prey for a variety of predatory invertebrates who are usually equipped with the jaws or mandibles, e.g., predatory rotifers (*Asplanchna*), cyclopoid copepods (*Mesocyclops*), predatory cladocerans (*Leptodora*), and midge larvae (*Chaoborus*)^20^. It makes logical sense, therefore, that ultrastructural defenses in rotifers could be an effective grazing deterrent toward multiple predatory invertebrates. Moreover, development of ultrastructural defenses may be particularly favored by small-sized rotifer prey species, which are not large enough to offer a ‘size refuge’. By now, predator-induced ultrastructural defenses in rotifers have received little attention. Determining the distribution of such defenses across different predator-prey systems will enhance our understanding of the ecological consequences of inducible defenses which evolve in a complex and multi-predator environment.

The herbivore rotifer *B*. *calyciflorus* and *B*. *angularis* and their predator *A*. *brightwellii* are often sympatric and widely distributed in ponds, lakes, and rivers all over the world^21–24^. The large *B*. *calyciflorus* can develop elongated spines in response to kairomones of *A*. *brightwellii* ^13^; however, the smaller *B*. *angularis* does not exhibit such inducible defenses. The present study compares the morphological responses of *B*. *calyciflorus* and *B*. *angularis* to *A*. *brightwellii* in terms of lorica ultrastructure, including thickness and hardness. However, the responses may be more evident in small-sized *B*. *angularis*. Ultrastructural defenses are often predicted to be adaptive^18, 19^, but direct evidence demonstrating this is relatively scarce. Here we evaluate whether the induced traits of *Brachionus* act as a defense against *Asplanchna*. Moreover, we anticipate finding some fitness costs associated with development of ultrastructural defenses in *Brachionus* because one of the prerequisites for evolution of inducible defenses is the significant cost which prevents defenses from being fixed^4^. Lastly, we assess the fitness consequences to *B*. *calyciflorus* and *B*. *angularis* in two prey-one predator interactions.

## Results

### Morphological responses of Brachionus to Asplanchna

**Figure 1:**
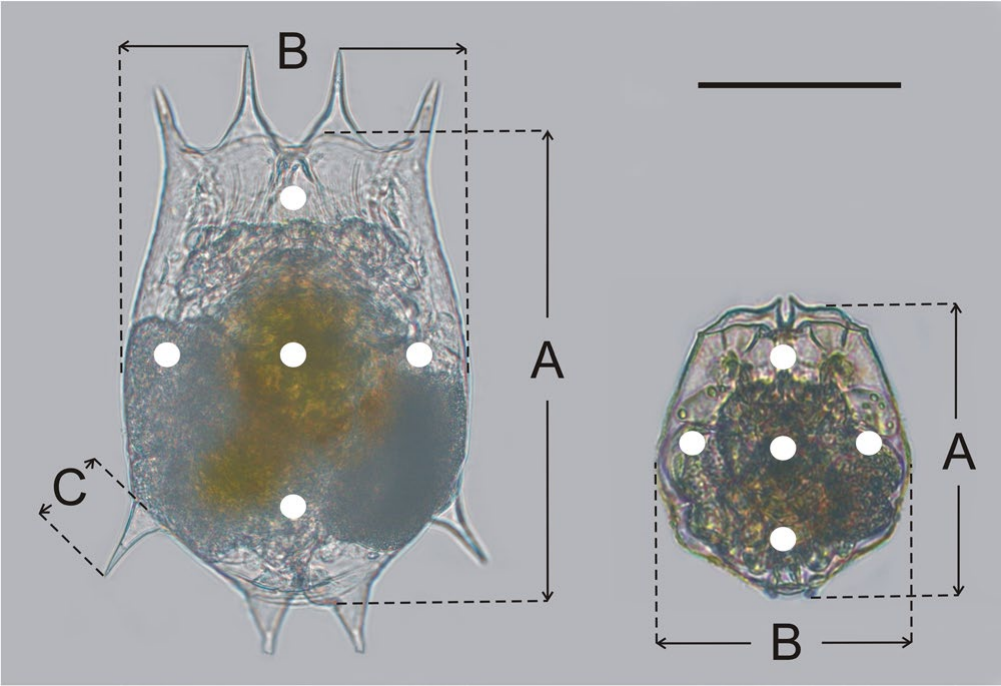
Biometric characters of *Brachionus calyciflorus* (left) and *Brachionus angularis* (right) measured in the present study. (**A**) body length; (**B**) body width; (**C**) length of posterolateral spine. White filled circles indicate the tested areas on each rotifer under atomic force microscopic observations. Scale bar = 100 µm.

The biometric characters of *Brachionus* measured in the present study were shown in Fig. 1. In response to *Asplanchna* kairomones, *B*. *calyciflorus* developed remarkably elongated posterolateral spines (general linear mixed-effects model, *F* = 749.869, *P* < 0.001) and larger body size (general linear mixed-effects model, *F* = 14.883, *P* = 0.001); however, *B*. *angularis* did not develop any spines and maintained body size (general linear mixed-effects model, *F* = 0.028, *P* = 0.869) when exposed to kairomones (Table 1). *Asplanchna* could induce ultrastructural defensive morphology in both *Brachionus* species; this defensive morphology was expressed as an increase in lorica thickness (supporting information, Fig. S1). Both *B*. *angularis* (general linear mixed-effects model, *F* = 53.889, *P* < 0.001) and *B*. *calyciflorus* (general linear mixed-effects model, *F* = 57.528, *P* < 0.001) developed thicker lorica when they were exposed to *Asplanchna* kairomones (Table 1). *B*. *angularis* has many minute projections spread throughout the lorica, but such is not the case in *B*. *calyciflorus* (supporting information, Fig. S2). These characteristics could explain the smooth lorica surface of *B*. *calyciflorus* and the uneven lorica surface of *B*. *angularis* in transverse section view (Fig. S1). In accordance with lorica thickness, *Asplanchna* kairomones also affected the strength of the lorica surface (Table 1). Observations using atomic force microscopy (AFM) showed that *B*. *angularis* exposed to kairomones obtained higher cuticle hardness than unexposed ones (general linear mixed-effects model, *F* = 695.110, *P* < 0.001), whereas the unexposed *B*. *calyciflorus* had lower cuticle hardness than exposed ones (general linear mixed-effects model, *F* = 132.943, *P* < 0.001). The data of force-depth indentation curves confirmed the above mentioned results (supporting information, Fig. S3), indicating that the AFM tip was pushed much deeper into the lorica of the unexposed *Brachionus* prey than that of kairomones-exposed prey.

**Table 1:** Biometric characters of *Brachionus calyciflorus* and *Brachionus angularis* cultured in the medium with and without *Asplanchna* kairomones.

	*B. calyciflorus*		*B. angularis*	
	K^−^	K^+^	K^−^	K^+^
**Visible morphological shifts**				
Body length (µm)	252.25 ± 1.38	255.92 ± 1.10	117.50 ± 0.76	121.65 ± 1.70
Body width (µm)	211.92 ± 12.32	229.42 ± 1.92	108.33 ± 2.32	115.79 ± 2.58
Length of posterolateral spine (µm)	49.16 ± 0.69	128.17 ± 0.55	—	—
Body size (**×**10^6^ µm^3^)	9.02 ± 1.03	10.60 ± 0.13	1.09 ± 0.06	1.29 ± 0.07
**Hidden morphological shifts**				
Lorica thickness (nm)	117.95 ± 12.79	182.44 ± 28.33	335.29 ± 28.72	472.46 ± 29.91
Lorica hardness (mean Young’s modulus)	1.54 ± 0.08	2.21 ± 0.07	3.28 ± 0.05	7.26 ± 0.17

Data are mean ± S.E. based on five replicated populations. K^−^ = rotifer culture medium without *Asplanchna* kairomones. K^+^ = rotifer culture medium with *Asplanchna* kairomones at a concentration of 100 *Asplanchna* L^−1^ with 24 h exposure.

### Benefits and costs of inducible defenses in Brachionus

**Figure 2:**
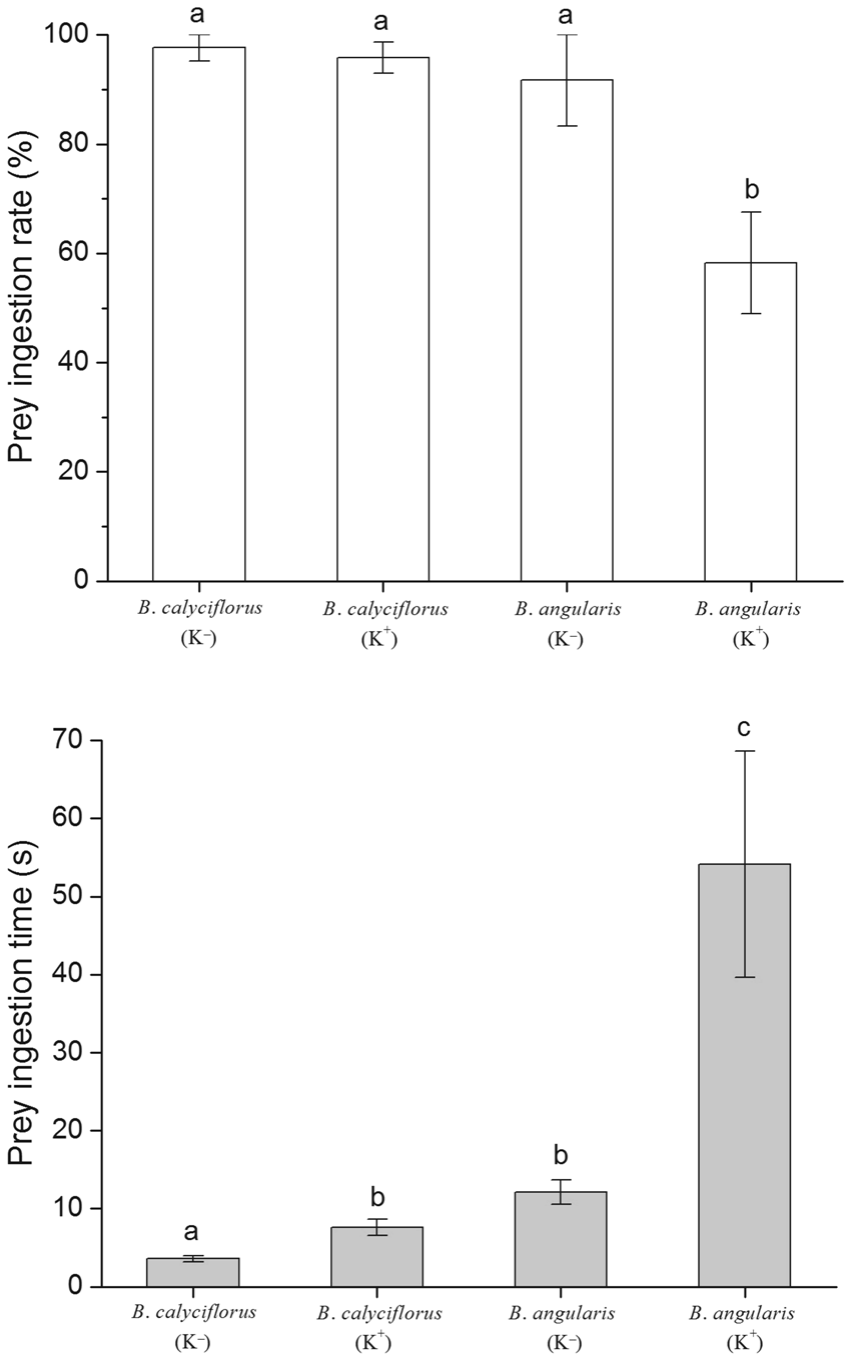
Prey ingestion rate (the percentage of the number ingested to number of successful captures) and prey ingestion time (time from the successful capture of prey by jaw to ingestion of captured from mastax to stomach) of *Asplanchna brightwellii* fed *Brachionus calyciflorus* (*B*.*c*.) and *Brachionus angularis* (*B*.*a*.) cultured in the medium with (K^+^) and without (K^−^) *Asplanchna* kairomones. To minimize the potential influences of body size and presence of attached eggs on feeding preference of *A*. *brightwellii*, we conducted the experiments with newborn *B*.*c*. (<1 h old) and non-ovigerous adult *B*.*a*. (>24 h old). Body length of *B*.*c*. in K^−^ = 137.0 ± 1.2 µm (n = 30); Body length of *B*.*c*. in K^+^ = 150.8 ± 1.4 µm (n = 30); Body length of *B*.*a*. in K^−^ = 124.6 ± 1.2 µm (n = 30); Body length of *B*.*a*. in K^+^ = 122.3 ± 1.4 µm (n = 30). Prey ingestion rate = 100 × (number of ingestion/number of successful capture); Prey ingestion time = time from successful capture of prey by jaw to ingestion of captured from mastax to stomach. Data of prey ingestion rate are mean ± S.E. based on 15 replicated observations within 10 min. Data of prey ingestion time are mean ± S.E. based on 15 replicated recordings.

The predatory *A*. *brightwellii* exerted disparate selectivity and preference on induced and noninduced rotifer prey (one-way ANOVA, *df* = 3, *F*_ingestion rate_ = 8.649, *P*_ingestion rate_ < 0.001, *F*_ingestion time_ = 10.257, *P*_ingestion time_ < 0.001, Fig. 2). The feeding behavior of *Asplanchna* indicated that the ingestion rate (percentage of ingested individuals after captured) for defended *B*. *angularis* was significantly lower than that of the three other rotifer morphotypes (Duncan *post hoc* tests, *P* < 0.05, Fig. 2). Approximately 40% of captured induced *B*. *angularis* were rejected unharmed by *Asplanchna*; however, almost all captured individuals of the three other rotifer morphotypes were ingested by *Asplanchna* (Fig. 2). The ingestion time (time from the successful capture of prey by jaw to ingestion of captured from mastax to stomach) reached the highest value (54.1 s) for defended *B*. *angularis* and the lowest value for undefended *B*. *calyciflorus* (3.6 s) but did not differ between undefended *B*. *angularis* and defended *B*. *calyciflorus* (Duncan *post hoc* tests, *P* < 0.05, Fig. 2).

**Figure 3:**
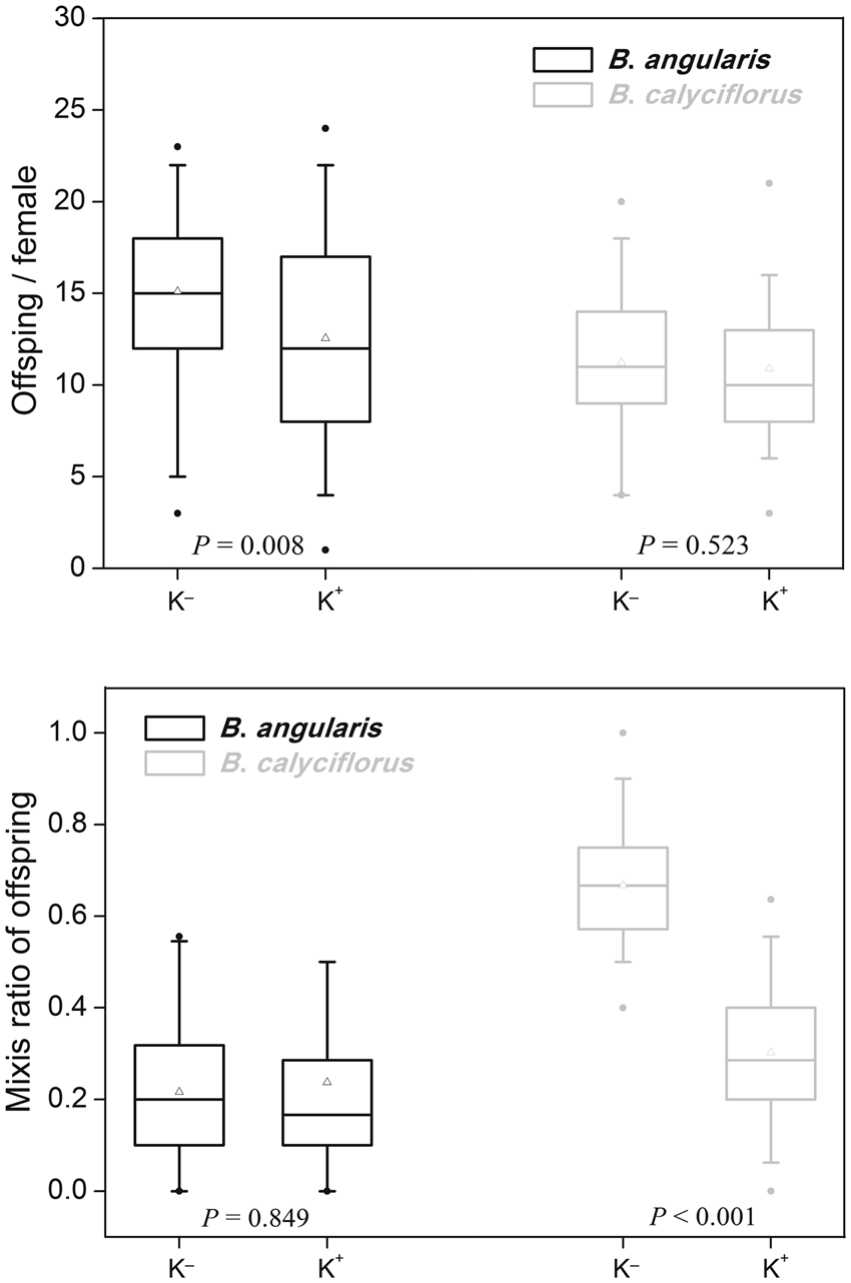
Boxplots of production and mixis ratio of offspring in *Brachionus calyciflorus* and *Brachionus angularis* cultured in the medium with (K^+^) and without (K^−^) *Asplanchna* kairomones. The solid horizontal line is the median and the triangle is the mean. The deviation bars represent the 5^th^ and 95^th^ percentiles, whereas the dots show the 1^th^ and 99^th^ percentiles. Data shown are based on 64 replicated *Brachionus* mothers.

We detected significant fitness costs of inducible defenses in two *Brachionus* species, but occurring in different forms (Fig. 3). Compared with unexposed *B*. *angularis*, production of offspring in exposed *B*. *angularis* decreased significantly (Mann-Whitney *U*-test, *Z* = −2.666, *P* = 0.008). However, no variation of reproduction between *B*. *calyciflorus* individuals cultured in K^+^ and K^−^ environments (Mann-Whitney *U*-test, *Z* = −0.639, *P* = 0.523) was observed. The mixis ratio of offspring in relation to kairomones showed a reverse trend in two *Brachionus* species (Fig. 3), indicating that those with *Asplanchna* kairomones had a lower mixis ratio of offspring in *B*. *calyciflorus* (Mann-Whitney *U*-test, *Z* = −9.175, *P* < 0.001), but showed no difference in *B*. *angularis* (Mann-Whitney *U*-test, *Z* = −0.191, *P* = 0.849).

### Fitness consequences for Brachionus when interacting with Asplanchna

**Figure 4:**
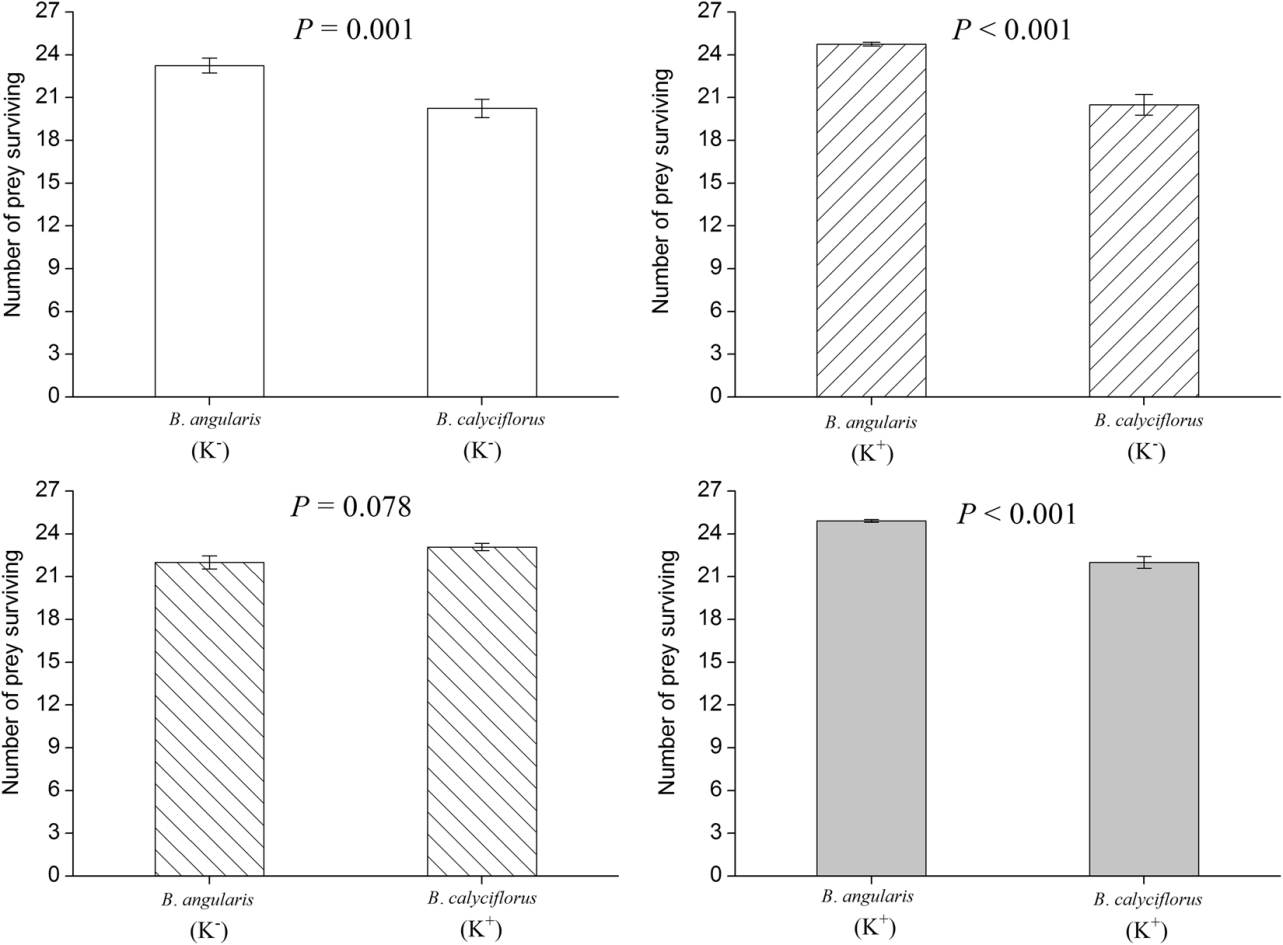
Survival of *Brachionus calyciflorus* (*B*.*c*.) and *Brachionus angularis* (*B*.*a*.) cultured together with *Asplanchna brightwellii*. At each prey combination, *B*.*c*. and *B*.*a*. cultured in the medium with (K^+^) and without (K^−^) *Asplanchna* kairomones are provided with proportion of 50%: 50% (25 individuals from each species). We conducted the experiments with newborn *B*.*c*. and non-ovigerous adult *B*.*a*. (see the legend in Fig. 3 for the detailed explanation). Data are mean ± S.E. based on 12 replicated observations.

In most cases, when *B*. *angularis* and *B*. *calyciflorus* were cultured together with adult *A*. *brightwellii*, *B*. *angularis* obtained much higher short-term fitness than *B*. *calyciflorus* (Fig. 4). Regardless of morphotypes, *B*. *angularis* individuals constantly had a higher survival than *B*. *calyciflorus* individuals (Mann-Whitney *U*-tests, *Z* = −3.085 ~ −4.330, *P* < 0.01) except for the combination of induced *B*. *calyciflorus* and non-induced *B*. *angularis*, which showed no difference in survival (Mann-Whitney *U*-test, *Z* = −1.862, *P* = 0.078).

**Figure 5:**
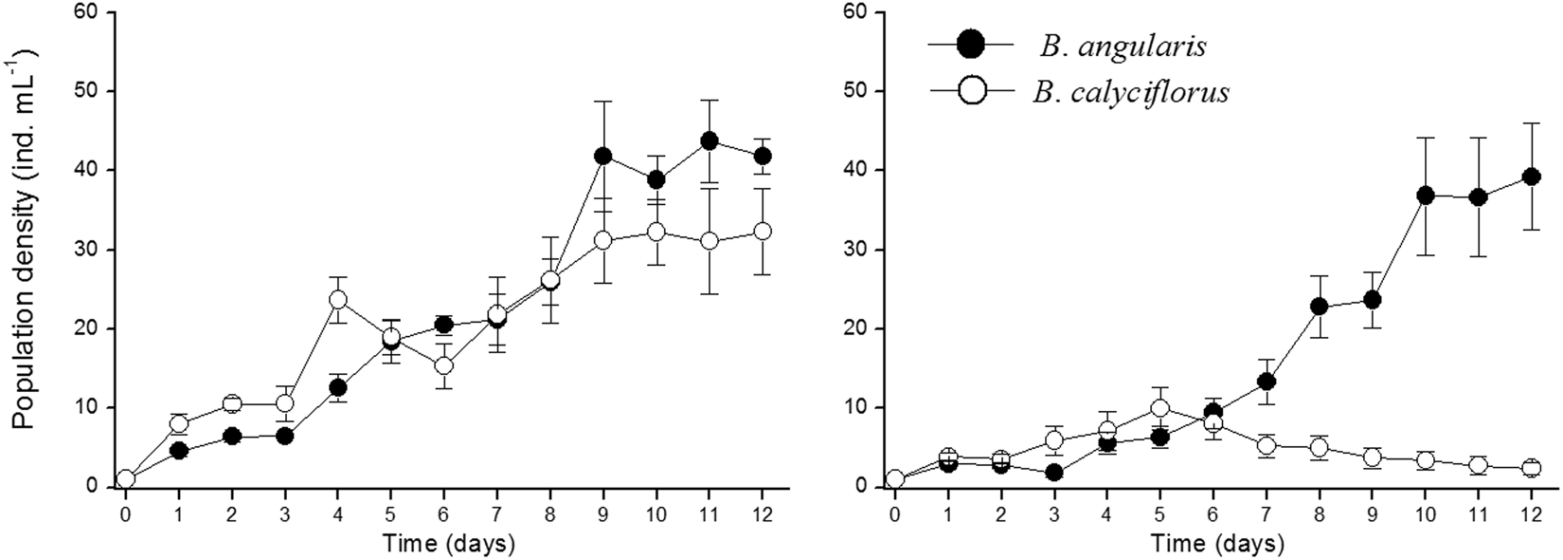
Population dynamics of *Brachionus angularis* (*B*.*a*.) and *Brachionus calyciflorus* (*B*.*c*.) fed with *Chlorella pyrenoidosa* at a concentration of 1 mg C L^−1^. Left: *B*.*a*. and *B*.*c*. were co-cultured without predator. Right: *B*.*a*. and *B*.*c*. were co-cultured under predation risk (*Asplanchna brightwellii* = 100 ind. L^−1^). Data shown are mean ± standard error values based on six replicates.

The population dynamics of *B*. *angularis* and *B*. *calyciflorus* in coexisting cultures with or without *A*. *brightwellii*, are shown in Fig. 5. The dynamics of the two *Brachionus* populations showed no difference during the culture period when cultured together (repeated measures ANOVA, *F*_1,10_ = 0.257, *P* = 0.623, Fig. 5). When the coexisting *Brachionus* were cultured together with adult *A*. *brightwellii*, population dynamics of the two *Brachionus* populations had no difference during the first half of coexistence experiments (repeated measures ANOVA, *F*_1,10_ = 0.871, *P* = 0.373) but differed significantly during the second half of coexistence experiments (repeated measures ANOVA, *F*_1,10_ = 28.162, *P* < 0.001). The presence of predatory *Asplanchna* resulted in near extinction of *B*. *calyciflorus* from the environments by the end of the experimental period (Fig. 5).

## Discussion

In our study we detected the presence of ultrastructural defenses in herbivorous *B*. *angularis* and *B*. *calyciflorus*, which are induced by the kairomones of the predatory rotifer *A*. *brightwellii* and manifested as increased lorica thickness and enhanced lorica hardness. *Asplanchna* can induce not only the development of longer posterolateral spines and larger body size in *B*. *calyciflorus*, but also shifts in the ultrastructure of lorica. However, *B*. *angularis* exhibits evident ultrastructural defenses in response to *Asplanchna* kairomones, but retains body shape. For *B*. *angularis* or *B*. *calyciflorus*, defended morphs are less susceptible to predation by *A*. *brightwellii* than basic morphs in terms of ingestion rate and ingestion time. Given the hard armor of defended *B*. *angularis*, individuals were physically better protected against predation, leading to higher fitness in predator-prey interactions. Compared with that of *B*. *angularis*, the elongated spine and strengthened lorica of induced *B*. *calyciflorus* provided less anti-predation capability. As a consequence of the development of inducible defenses, both *B*. *angularis* and *B*. *calyciflorus* experienced fitness costs; however, these costs were manifest in different forms. The costs of inducible defenses were expressed as decreased reproduction in *B*. *angularis* and reduced sex investment in *B*. *calyciflorus*.

Shifts of morphology, life history, and behavior in response to predatory vertebrates or invertebrates have been reported as inducible defenses in cladocerans^5^. However, the main inducible defenses in rotifers are morphological, including development or elongation of spines^10^. The exuberant phenotypes provide effective protection from small predatory invertebrates (e.g. *Asplanchna*)^25, 26^. This protection can be explained by the ‘anti-lock-and-key’ hypothesis^27^. However, defensive morphologies with spination might be ineffective against large predatory invertebrates, which are always equipped with strong mandibles^25, 28^. For defense against large predatory invertebrates, the development of hard armor would be more adaptive. Although both *Brachionus* prey species exhibit the combination of a harder and thicker lorica under predation risk, such inducible defenses are more evident and effective in the smaller sized *B*. *angularis*. The types of inducible defenses employed by prey species depend on the evolutionary history and the ecological environment of the organism^29, 30^. On the one hand, multi-predator environments are common in nature^30, 31^. The coexistence of a variety of predatory invertebrates results in heavy predation on rotifer prey species, particularly small rotifers^20^. On the other hand, spined morphs of small rotifers may still be not large enough to offer ‘size refuge’ from predation. Therefore, we hypothesize that the evolution of ultrastructural defenses may be more common in small rotifer species, and is adaptive to multiple predatory invertebrates. Small rotifer prey may be easily seized and swallowed whole by predatory invertebrates, but an induced rigid lorica can protect them from being completely ingested. Moreover, differences in lorica thickness and hardness will increase individual fitness without altering body shape, thereby avoiding changes in hydrodynamics (e.g., shifts in center of gravity)^32^ and graspability (e.g., loss of relatively smooth cuticle due to spination)^33^. These speculations are possible because some small but hard-armored rotifers (e.g., *B*. *budapestinensis* and *K*. *cochlearis*) are less vulnerable to predatory invertebrates^20, 34^.

In freshwater ecosystems, two types of defense strategies are recognized: (1) pre-capture defenses that enable prey to avoid observation or detection by a predator and (2) post-capture defenses that protect prey from capture and ingestion by a predator^1^. In our work, inducible ultrastructural defenses in *Brachionus* can be categorized as post-capture defenses, in which induced individuals with greater rigidity of their lorica are capable of impeding handling and ingestion by *Asplanchna*. Our results verify the general idea that architectural or ultrastructural defenses of prey species provide effective mechanical protection against many potential predators^18, 35^. Based on our data, we can further predict that rotifers with harder lorica will be less susceptible to a variety of predatory invertebrates because such ultrastructural defenses make prey difficult for predatory invertebrates to crush and ingest. Although development of a harder lorica together with long spines and large sizes may further impede capture or handling of *B*. *calyciflorus* by predator, the well-defended *B*. *angularis* obtains high short-term and long-term fitness when they coexist with *A*. *brightwellii*. Some may argue that the experimental designs with newborn *B*. *calyciflorus* in predation experiments may underestimate the performance of strengthened lorica and size effects of *B*. *calyciflorus* populations in defending against predation from *Asplanchna*. However, we argue that similar characteristics of the lorica may exist in both neonate and adult *B*. *calyciflorus*, because empirical data indicated that the handling time of prey for *A*. *brightwellii* did not differ between young and adult spined *B*. *calyciflorus* ^25^. Moreover, a range of sizes of *Brachionus* would coexist with the predator in long-term population experiment. Finally, *B*. *angularis* obtained higher survival in both short-time and long-time cultures with the predator. Thus the potential effects of varied sizes could be minimized, and prey with thicker and harder lorica would be better defended.

Commonly, resource competition between identical rotifer species causes the extinction of the inferior competitor^36^. However, some experimental evidences showed that inducible defenses could promote coexistence of identical species when they competed for a single food source^37^. The costs of inducible defenses create negative feedback loops that prevent strong population fluctuations under predation risk^8^. Our data verify this idea, showing that two *Brachionus* coexisted in the environments containing predator kairomones. In addition, the preferential selection of prey species by a common predator as a result of the disparity of prey availability and prey palatability can drive more susceptible species to extinction^9, 38–40^. Two *Brachionus* prey species have developed inducible defenses in response to *Asplanchna* kairomones, but the well-defended *B*. *angularis* obtains higher fitness when they coexist with *A*. *brightwellii*. Although *B*. *calyciflorus* was not completely outcompeted during the course of the experiment, its density was marginal in comparison with the superior competitor *B*. *angularis*. Considering that the intensity of predation and effectiveness of inducible defense frequently play vital roles in predator-prey interactions^39, 40^, future studies associated with influences of inducible defenses on multi-trophic communities should consider these ecological factors in their theoretical or empirical models.

Allocation costs in defended rotifers have been hypothesized and tested. Some empirical studies have supported this hypothesis^26, 41, 42^, whereas other research found no defense cost^25, 43^. Unsuccessful attempts to observe fitness costs in relation to inducible defense may be a consequence of the experimental design rather than the lack of tradeoffs^14, 44^. Here, we detected allocation costs, that is, decreased reproduction or reduced sexual reproduction, in defended *Brachionus* prey species, verifying the previous findings and proving the presence of fitness costs in rotifers^26, 41, 42^. In our work, neonates of the basic phenotypes are cultured in both K^+^ and K^−^ environments to assess costs of inducible defenses; thus, we may have underestimated the magnitude of cost in the two *Brachionus* species because we only evaluated the cost of producing defenses and neglected the cost of maintaining such defenses. In addition, we speculate that the maintenance costs of defended *B*. *angularis* will be much lower than that of *B*. *calyciflorus* because morphological changes can alter the hydrodynamics of *B*. *calyciflorus* and probably result in high energy requirements for maintenance. Interestingly, these two species have developed disparate allocation costs. For *B*. *calyciflorus*, inducible defenses cannot provide complete protection against predatory invertebrates^25^; accordingly, energy expenditure in sexual reproduction is saved and allocated to the production of more parthenogenetic offspring to offset predation loss^42^. In contrast, the relatively high survival rates of *B*. *angularis* compared with that of *B*. *calyciflorus* in the presence of predatory invertebrates may preserve the energy expenditure of sexual reproduction and maintain resting-egg production, which is important for the long-term fitness of the population^45^.

In conclusion, the results of this study confirmed the existence of predator-induced ultrastructural defenses, which are expressed as increased lorica thickness and enhanced lorica hardness, in *Brachionus* rotifers. Such inducible defenses are usually invisible under an optical microscope and are referred to as hidden defensive morphology. To our knowledge, this is the first time that the presence of inducible ultrastructural defenses in monogonont rotifers has been observed. Our results not only expanded the understanding of inducible ultrastructural defense to other planktonic taxa, which were previously only observed in cladocerans^15, 19^, but also verified the effective mechanical protection of such hidden defenses. Furthermore, our data suggest that the evolution of inducible ultrastructural defenses in prey populations may be adapted to a variety of predatory invertebrates. Hidden ultrastructural defenses are supposed to be taxonomically widespread^30^; thus, further empirical and field experiments should be designed to uncover the ecology and evolution of such inducible defenses in other rotifers and other planktonic taxa.

## Methods

### Rotifer culture

The herbivores *B*. *angularis* and *B*. *calyciflorus* and carnivorous *A*. *brightwellii* were obtained from wetting of dried sediment collected from a freshwater pond (39°57′N; 116°21′E) in Beijing, China, following the methods of previous studies^42, 46^. One newly hatched female *Brachionus* or *Asplanchna* was isolated and allowed to reproduce parthenogenetically in the COMBO medium^47^ enriched with 40 ml L^−1^ soil water extract^40^ (hereafter COMBO). *Brachionus* was cultured with the food alga *Chlorella pyrenoidosa*, which was also cultured in COMBO, at a concentration of 1.0 mg C L^−1^. *Asplanchna* was fed with a mixture of *B*. *angularis* and *B*. *calyciflorus* at a density of 10 ind. mL^−1^. The culture medium was renewed every two days, and the rotifer densities were maintained at low levels (≈200 ind. L^−1^). All rotifer and algal cultures, as well as experimental incubation conditions, were maintained at 20 ± 1.0 °C with a 14 L:10 D photoperiod. Before use in the experiments, all *Brachionus* and *Asplanchna* rotifer clones were cultured in the laboratory for at least a month in exponential growth phase.

### Morphological responses of Brachionus to Asplanchna

The experiment was performed in 1.0 L glass beakers containing 0.5 L COMBO with (K^+^) or without (K^−^) *Asplanchna* kairomones. The preparation of *Asplanchna*-conditioned COMBO was described in previous studies^42, 46^. In brief, 100 adult females of *A*. *brightwellii* (≈800–900 µm in length) were placed into a 1000 mL COMBO and deprived of food supply for 24 hours. Then, conditioned COMBO was filtered with fiber filters (0.45 µm). The predation risk of *Asplanchna* in the present work is relatively high (100 ind. L^−1^), but this is common in natural water environments^21, 48, 49^. The experiment was started by placing 10 egg-carrying amictic females of *B*. *angularis* or *B*. *calyciflorus* into beakers containing K^+^ or K^−^ COMBO. *Brachionus* were fed daily with 1.0 mg C L^−1^ of *C*. *pyrenoidosa*. The culture medium was renewed every day and the density of *Brachionus* population was controlled below 500 ind. L^−1^. The density of rotifer prey was relatively low, which would produce the maximum magnitude of defense in response to *Asplanchna* kairomones^50^. *Brachionus* are fast-growing monogonont species with the juvenile development time less than 24 h at 20 °C^51^. Furthermore, life span of experimental clones was less than 7.5 days in this study (supporting information, Table S1). Therefore, our experiments lasted for 15 days that can diminish the potential maternal effects on inducible defenses^46^. Then, adult *B*. *angularis* or *B*. *calyciflorus* was harvested and preserved in 70% ethanol to measure the thickness and hardness of lorica. For each *Brachionus* species, five replicates in each treatment (K^+^ or K^−^) were conducted.

Transmission electron microcopy was used to evaluate lorica thickness of *Brachionus*. *Brachionus* individuals from each replicate were fixed in 2.5% glutaraldehyde for 2 h. Following three 20-min changes in 0.1 M phosphate buffer (pH 7.5), rotifers were post-fixed at 4 °C in 1% osmic acid (2 h), dehydrated in a graded ethanol series (50%, 70%, 80%, 90%, and 100%), and embedded in epoxy resin. Sections were cut with an ultratome (E + E Elektronik), mounted on grids, post-stained with 2% aqueous uranyl acetate (30 min) and lead citrate (15 min), and examined under an electron microscope (JEM-1200EX, JEOL). Microscopic images of transverse sections were used to measure lorica thickness. Micrographs of five different areas in a transverse section were taken at 10,000× magnification. For each replicate, three random transverse sections, belonging to three different rotifer individuals, were assessed. Based on the scale bar in the micrograph, we measured the lorica thickness with Auto CAD 2014 software. The value of lorica thickness in each replicate was based on the mean of the three samples. It should be noticed that we could not locate the sections used for the measurement of thickness in a certain part of *Brachionus* body due to their small body size. Hence, the influences of varying thickness in the different parts of body might not be excluded in the present work.

An atomic force microscope (MultiMode 3D, Veeco, USA) with a 125 × 125 µm lateral scan range and a 125-µm z-extension stage was used to evaluate lorica hardness of *Brachionus*. A microscope (Olympus SZ2-STS, 9F07795), connected to CCD camera (10x-A, Nikon 1102716), allowed precise positioning of the silicon nitride cantilever at the area of interest on the lorica. The silicon nitride cantilevers (14–26 KHz, SNL-10, Veeco, USA) with a nominal spring constant of 0.32 N/m and a pyramid tip (nominal tip radius = 2 nm) were used for all atomic force microscopy (AFM) measurements. A 9 × 9 nm^2^ force map containing one measurement point was probed and a force-depth indentation curve was obtained from each area of interest. Five different areas were analyzed on each rotifer (Fig. 1), and two randomly selected rotifers were measured per replicate. The maximum force exerted on the lorica was set to 3 nN, resulting in maximum indentation depth of approximately 15 nm, which ensured that only the hardness of the lorica surface was detected. The Young’s modulus (E) used to indicate the hardness *E* = *F*×(1 −  *v* ^2^)/(0.7453 × *δ* ^2^ × tan*α*), from the force-depth indentation curve with the equation described in previous works^15, 19^:

$$E=F\times (1-{v}^{2})/(0.7453\times {\delta }^{2}\times \,\tan \,\alpha ),$$

where E is the Young’s modulus, F is the indentation force applied to the sample, *v* is the Poisson’s ratio (set to 0.5 in this study), *δ* is the indentation, and *α* is the face angle of the pyramid (22.5° in this study). In each case, AFM was measured using a wet rotifer sample. The mean Young’s modulus of each rotifer was calculated to indicate the average hardness of lorica, and the value of lorica hardness in each replicate was based on the mean of the two samples.

We also evaluated the visible morphological changes of *Brachionus* in response to *Asplanchna* kairomones, e.g., spine development and elongation, and body size changes. Six *Brachionus* were randomly chosen from the preserved samples of each replicate. Biometric characteristics, such as body length and width and length of developed or elongated spines (e.g. posterolateral spines), of *Brachionus* rotifers were measured to the nearest 2.5 µm at a magnification of 400× (Fig. 1). Body size (volume) of *Brachionus* was calculated following the previous work^52^. The values of biometric characters in each replicate were based on the mean of the six samples.

### Benefits and costs of inducible defenses in Brachionus

When exposed to *Asplanchna* kairomones, both *Brachionus* species developed morphological or ultrastructural defenses (see ‘Results’ for detailed information). Thus, we assessed the performance of inducible defenses of *B*. *angularis* and *B*. *calyciflorus* in defending against *A*. *brightwellii* predation. Following the methods described above, we obtained populations of *B*. *angularis* and *B*. *calyciflorus* in the K^+^ and K^−^ environments. Defense efficiency was determined based on *Asplanchna* ingestion rate (the percentage of the number ingested to number of successful captures) and *Asplanchna* ingestion time (time from the successful capture of prey by jaw to ingestion of captured from mastax to stomach). The criteria used to record the feeding behavior of *Asplanchna* were detailed in previous studies^53^. The main goal of this work was to evaluate the effective mechanical protection resulting from variations of lorica ultrastructure. Given the body size and oviposition of rotifers can influence the feeding behavior of *Asplanchna* ^25, 53^, we conducted the experiments with newborn *B*. *calyciflorus* (<1 h old) and non-ovigerous adult *B*. *angularis* (>24 h old) to minimize the potential influences of body size and presence of attached eggs on feeding preference of *A*. *brightwellii*. The detailed information of biometric characters of newborn *B*. *calyciflorus* and non-ovigerous adult *B*. *angularis*, which were measured based on the methods mentioned above, was shown in Fig. 3.

One starved adult *Asplanchna* (6-h starvation) was introduced into each cell of a 24-well plate containing 0.5 mL COMBO and 10 individuals of one particular herbivore rotifer species cultured in K^+^ or K^−^. The number of captured and ingested prey within 10 min was recorded with a stereomicroscope. In a separate experiment, ingestion time (handling time) of starved adult *Asplanchna* when they were supplied with the same types of rotifer prey was recorded. When an *Asplanchna* ingested one prey item, it was replaced by another starved *Asplanchna*, and the number of rotifer prey was restored to 10 individuals per 0.5 mL. The ingestion time of *Asplanchna* was recorded to the nearest 0.1 s using a stopwatch on a smart-phone. For each of the prey species from either K^+^ or K^−^, a total of fifteen replicated observations were conducted to measure ingestion rate and ingestion time.

At present, two forms of costs in association with inducible defenses have been reported in monogonont rotifers: decreased reproduction and reduced sex investment^26, 42^. In the present work, we conducted life table studies to determine the costs of inducible defenses of *B*. *angularis* and *B*. *calyciflorus*. A juvenile amictic female (<6 h old) *B*. *angularis* or *B*. *calyciflorus* which was cultured in K^−^ environment was placed into each cell of a 24-well plate containing 1.0 mL freshly prepared K^+^ and K^−^ COMBO with 1.0 mg C L^−1^ of *C*. *pyrenoidosa*. The kairomone concentration in the environment and the method to prepare K^+^ COMBO were the same as mentioned above. For each species-environment treatment combination, a total of 64 replicated females were evaluated. The culture medium in each treatment was renewed every 12 h. During this process, the number of newborn *B*. *angularis* and *B*. *calyciflorus* in each replicate was counted and then the newborns were transferred individually to 24-well culture plates containing 1 mL COMBO and 1.0 mg C L^−1^ *C*. *pyrenoidosa*. These newborn rotifers were cultured until they became ovigerous adults and were then classified as amictic (female-producing) female or mictic (male-producing) female. Experiments were terminated when all mother rotifers died. In cyclical parthenogens such as brachionids, sex investment is frequently defined as the production of male-producing females^54, 55^. Thus, mean number of offspring and the average mixis ratio of offspring (percentage of male-producing females) were used to assess the costs of inducible defenses in the two rotifer prey species.

### Fitness consequences for Brachionus when interacting with Asplanchna

In this work, we evaluated fitness consequences of inducible defenses for *B*. *angularis* and *B*. *calyciflorus* to the presence of *A*. *brightwellii* based on short-term (40 min) and long-term (12 days) multispecies interactions. In the short-term studies, the susceptibility of the basic and induced morphs of *B*. *angularis* and *B*. *calyciflorus* to predation by *A*. *brightwellii* was assessed. Here again, we conducted short-term experiments with newborn *B*. *calyciflorus* (<1 h old) and non-ovigerous adult *B*. *angularis* (>24 h old). The combination of either 25 induced or 25 non-induced *B*. *calyciflorus* together with either 25 induced or 25 non-induced *B*. *angularis* was exposed to one starved adult *A*. *brightwellii* (6-h starvation) in each cell of a 6-well plate with 5.0 mL COMBO for 40 min. The *Asplanchna* was removed from the environment, and *Brachionus* populations in each combination were preserved with 2% formalin; then, the number of individuals of each *Brachionus* morph was recorded. Each experimental combination was run in 12 replicates.

In the long-term studies, coexistence experiments of the two *Brachionus* species with or without *Asplanchna* predation were performed. Experiment was conducted in each cell of a 6-well plate containing 10 ml COMBO and 1.0 mg C L^−1^ *C*. *pyrenoidosa*. The initial density of both *B*. *angularis* and *B*. *calyciflorus* in the coexistence experiment was 1 ind. mL^−1^. During the initiation of a separate coexistence experiment, we introduced one adult *A*. *brightwellii* into the environment to produce predation pressure. The COMBO used in coexistence experiments was conditioned with *Asplanchna* at a density of 100 ind. L^−1^ for a 24-h exposure. Every day, the culture medium was renewed with the appropriate algal food concentration, and the number of each *Brachionus* species in the environment was counted. During this period, newborn *Asplanchna* were removed from the environment, and dead *Asplanchna* were replaced with new ones. The experiments were terminated after 12 days when the trends of most populations became obvious and stable. Each experimental culture was run in six replicates.

### Data analysis

We performed a general linear mixed-effects model to test the effects of *Asplanchna* kairomones on visible and hidden morphological changes in *Brachionus*. A one-way ANOVA using treatment (morphotype) as the fixed factor was conducted to analyze the prey ingestion rate and prey ingestion time of *Asplanchna*. When significant differences (*P* < 0.05) were detected, Duncan’s test was used for pairwise comparisons. Nonparametric Mann-Whitney *U*-tests were performed to compare: (i) the mean number of offspring and average mixis ratio of offspring in life table studies, and (ii) the survival of rotifer prey cultured together with *Asplanchna* in short-term fitness evaluations. Repeated measures ANOVA were performed to compare the performance of *Brachionus* populations with and without predation in long-term fitness evaluations. Assumptions for ANOVA were evaluated using the Levene’s test for homogeneity of variances and the Kolmogorov-Smirnov test for normality. The data, expressed as percentage or ratio, were arcsine transformed. All statistical analyses were performed using the SPSS statistical package version 21.0.

## Electronic supplementary material

Supporting information
