# Supplementary material for: Hidden defensive morphology in rotifers: benefits, costs, and fitness consequences
Source: Sci Rep. 2017 Jul 3;7:4488. doi: 10.1038/s41598-017-04809-z (PMC5495802; doi:10.1038/s41598-017-04809-z)
Supplement: Supplementary file 1 — Supporting information [file 41598_2017_4809_MOESM1_ESM.pdf]

Supporting information

Hidden defensive morphology in rotifers: benefits, costs, and fitness consequences

Xuwang Yin*, Wen Jin, Yanchun Zhou, Peipei Wang, Wen Zhao

Liaoning Provincial Key Laboratory for Hydrobiology, College of Fisheries and Life Science, Dalian Ocean University, Dalian, China.

Table S1. Life span of *Brachionus calyciflorus* and *Brachionus angularis* cultured in the medium with and without *Asplanchna* kairomones. Data are mean ± S.E. based on 64 replicated *Brachionus* mothers. K^- = rotifer culture medium without *Asplanchna* kairomones. K^+ = rotifer culture medium with *Asplanchna* kairomones at a concentration of 100 *Asplanchna* L^{-1} with 24 h exposure.

	B. calyciflorus		*B. angularis*	
	K^-	K^+	K^-	K^+
Life span (d)	5.10 ± 0.17	5.14 ± 0.16	6.71 ± 0.18	7.07 ± 0.23

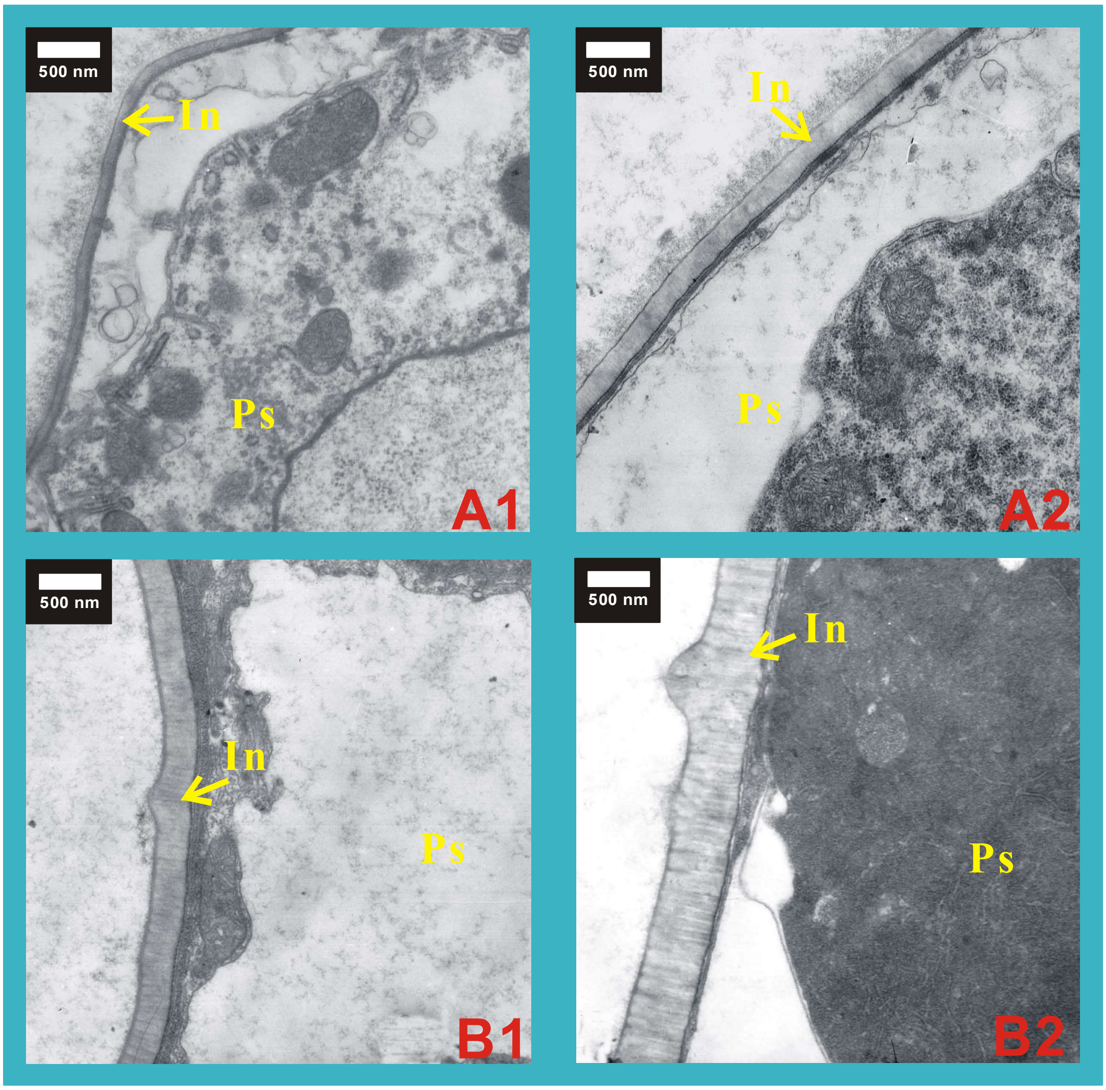

Figure S1. Transmission electron micrographs of body wall of *Brachionus calyciflorus* (*B.c.*) and *Brachionus angularis* (*B.a.*). A1 = *B.c.* in K^{-}. A2 = *B.c.* in K^{+}. B1 = *B.a.* in K^{-}. B2 = *B.a.* in K^{+}. In = integument (body wall); Ps = pseudocoelom (body cavity).

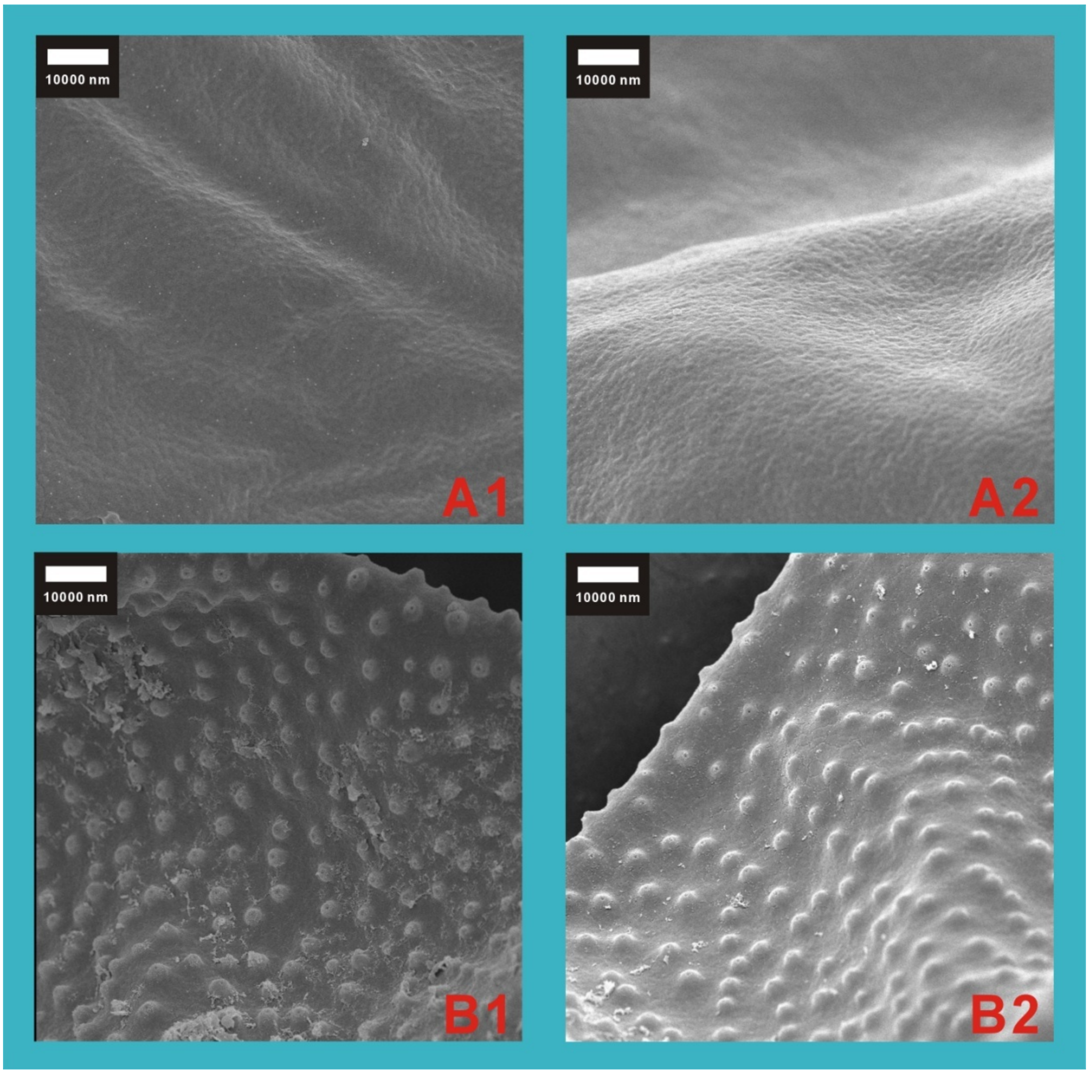

Figure S2. Scanning electron micrographs of lorica surface of *Brachionus calyciflorus* (*B.c.*) and *Brachionus angularis* (*B.a.*). A1 = *B.c.* in K^-. A2 = *B.c.* in K^+. B1 = *B.a.* in K^-. B2 = *B.a.* in K^+.

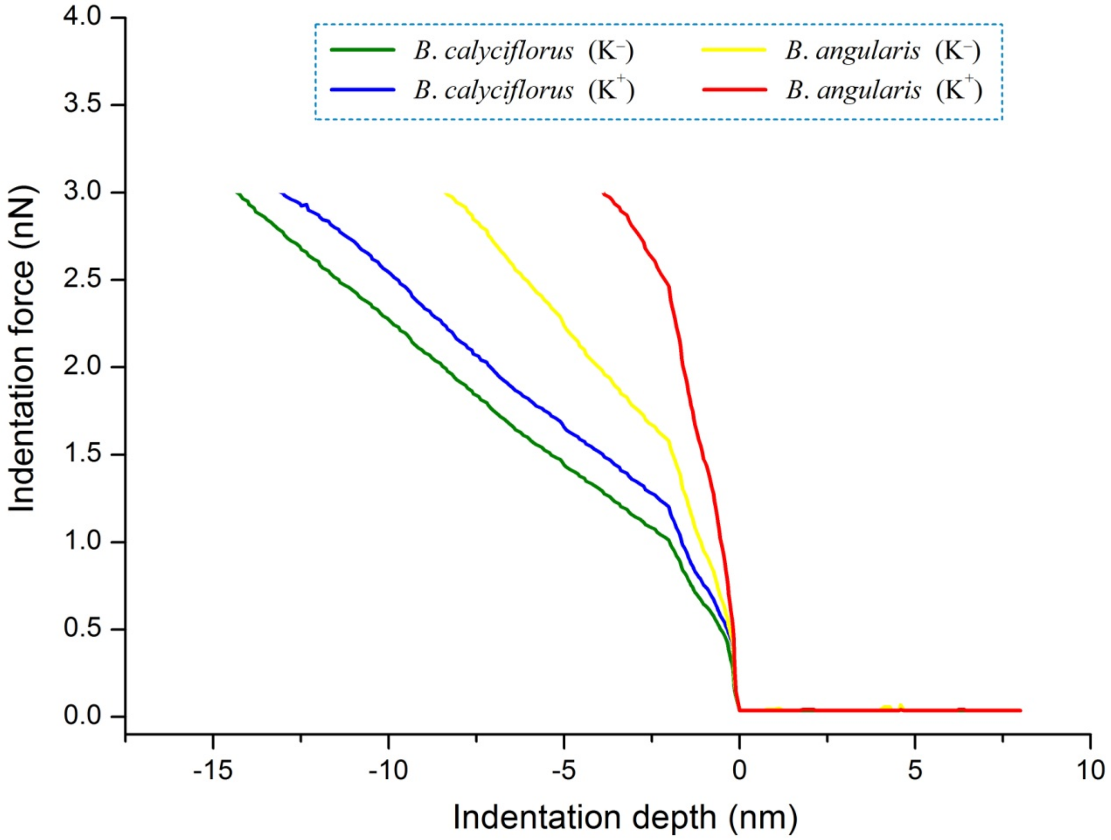

Figure S3. Comparison of force–depth indentation curves obtained from *Brachionus calyciflorus* and *Brachionus angularis* cultured in the medium with (K^+) and without (K^-) *Asplanchna* kairomones. Data shown are mean values based on five replicated populations. Standard error values (= 0.00001 ~ 0.02) are too small to be shown.
